# *N*′-[(*E*)-Benzyl­idene]-2-(6-meth­oxy­naphthalen-2-yl)propano­hydrazide

**DOI:** 10.1107/S1600536813026986

**Published:** 2013-10-05

**Authors:** Joel T. Mague, Mehmet Akkurt, Shaaban K. Mohamed, Mahmoud A. A. El-Remaily, Mustafa R. Albayati

**Affiliations:** a Department of Chemistry, Tulane University, New Orleans, LA 70118, USA; b Department of Physics, Faculty of Sciences, Erciyes University, 38039 Kayseri, Turkey; c Chemistry and Environmental Division, Manchester Metropolitan University, Manchester M1 5GD, England; d Chemistry Department, Faculty of Science, Mini University, 61519 El-Minia, Egypt; e Department of Chemistry, Faculty of Science, Sohag University, 82524 Sohag, Egypt; f Kirkuk University, College of Science, Department of Chemistry, Kirkuk, Iraq

## Abstract

The title mol­ecule, C_21_H_20_N_2_O_2_, exists in the solid state in the ‘extended’ form. The crystal packing consists of ribbons of mol­ecules extending parallel to *c* and associated *via* N—H⋯O and weak C—H⋯O hydrogen bonds. C—H⋯π inter­actions are also present.

## Related literature

For general clinical use of nonsteroidal anti-inflammatory drugs (NSAIDs) and Naproxen^®^, see: Merlet *et al.* (2013 ▶); Khanna *et al.* (2006 ▶); Bhaduri *et al.* (1995 ▶); Dharmani *et al.* (2004 ▶). For common side effects of NSAIDs, see: Neeraj *et al.* (2010 ▶); Asif (2009 ▶); Parmeshwari *et al.* (2009 ▶).
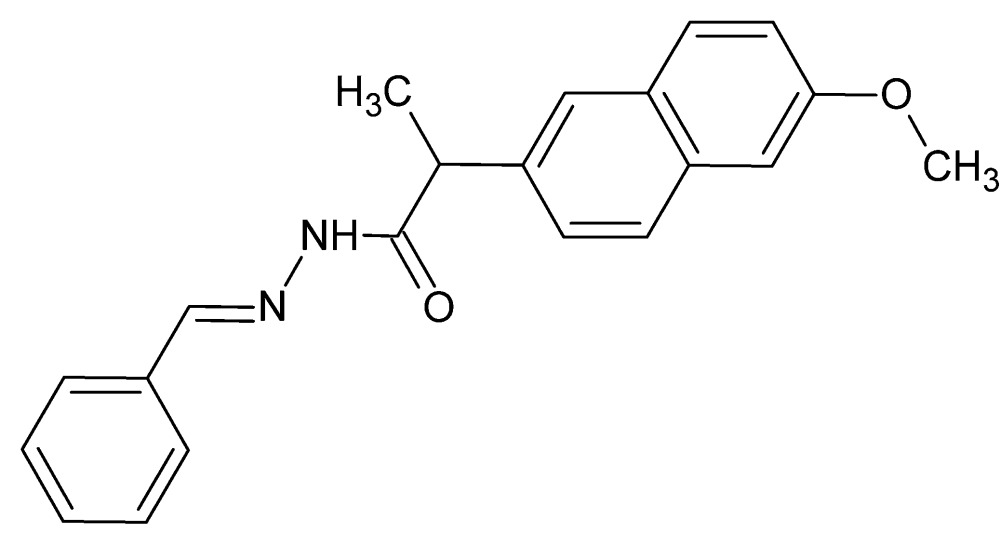


## Experimental

### 

#### Crystal data

C_21_H_20_N_2_O_2_
*M* *_r_* = 332.39
Orthorhombic, 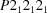

*a* = 10.3754 (17) Å
*b* = 32.519 (5) Å
*c* = 5.0615 (8) Å
*V* = 1707.7 (5) Å^3^
*Z* = 4
Mo *K*α radiation
μ = 0.08 mm^−1^
*T* = 150 K
0.15 × 0.11 × 0.11 mm

#### Data collection

Bruker SMART APEX CCD diffractometer
Absorption correction: multi-scan (*SADABS*; Bruker, 2013 ▶) *T*_min_ = 0.73, *T*_max_ = 0.99
28152 measured reflections
4230 independent reflections
3882 reflections with *I* > 2σ(*I*)
*R*_int_ = 0.053

#### Refinement

*R*[*F* ^2^ > 2σ(*F* ^2^)] = 0.052
*wR*(*F* ^2^) = 0.128
*S* = 1.10
4230 reflections
232 parameters
H atoms treated by a mixture of independent and constrained refinement
Δρ_max_ = 0.34 e Å^−3^
Δρ_min_ = −0.26 e Å^−3^

### 

Data collection: *APEX2* (Bruker, 2013 ▶); cell refinement: *SAINT* (Bruker, 2013 ▶); data reduction: *SAINT*; program(s) used to solve structure: *SHELXTL* (Sheldrick, 2008 ▶); program(s) used to refine structure: *SHELXL2013* (Sheldrick, 2008 ▶); molecular graphics: *ORTEP-3 for Windows* (Farrugia, 2012 ▶); software used to prepare material for publication: *WinGX* (Farrugia, 2012 ▶) and *PLATON* (Spek, 2009 ▶).

## Supplementary Material

Crystal structure: contains datablock(s) global, I. DOI: 10.1107/S1600536813026986/sj5356sup1.cif

Structure factors: contains datablock(s) I. DOI: 10.1107/S1600536813026986/sj5356Isup2.hkl

Supplementary material file. DOI: 10.1107/S1600536813026986/sj5356Isup3.cml

Additional supplementary materials:  crystallographic information; 3D view; checkCIF report

## Figures and Tables

**Table 1:** Hydrogen-bond geometry (Å, °) *Cg*2 and *Cg*3 are the centroids of the C4–C9 benzene and C16–C21 phenyl rings, respectively.

*D*—H⋯*A*	*D*—H	H⋯*A*	*D*⋯*A*	*D*—H⋯*A*
N1—H1⋯O2^i^	0.94 (3)	1.98 (4)	2.892 (3)	163 (3)
C15—H15⋯O2^i^	0.95	2.49	3.261 (3)	138
C18—H18⋯O1^ii^	0.95	2.59	3.209 (4)	123
C1—H1*C*⋯*Cg*3^iii^	0.98	2.91	3.696 (3)	138
C12—H12⋯*Cg*2^i^	1.00	2.78	3.661 (3)	147

Symmetry codes: (i) 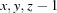; (ii) 
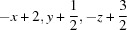
; (iii) 
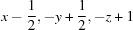
.

**Fig. 1.**
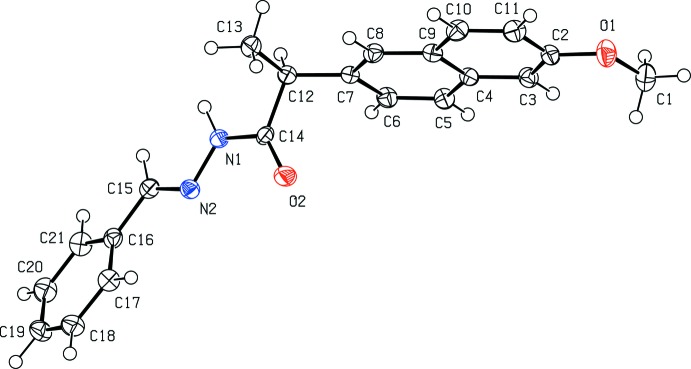
Perspective view of the title molecule with 50% probability displacement ellipsoids.

**Fig. 2.**
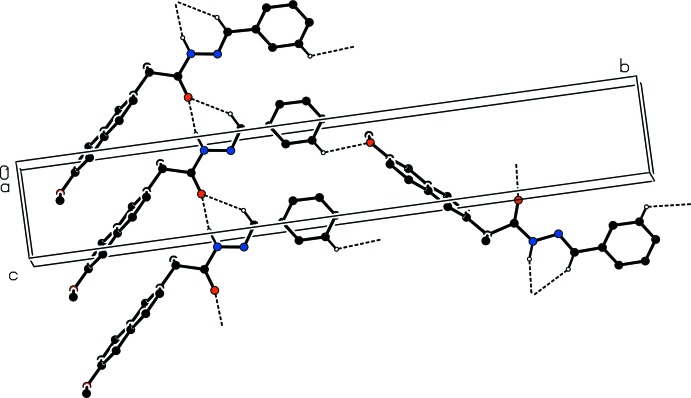
The hydrogen bonding (dotted lines) viewed along the a axis of the title compound.

**Fig. 3.**
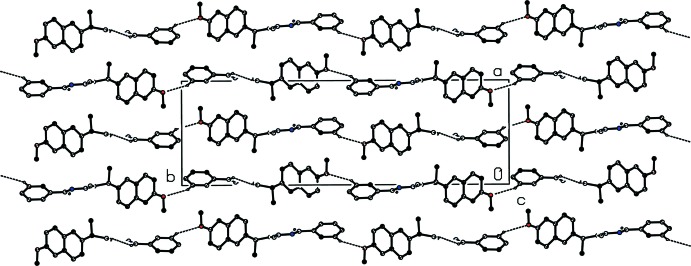
Packing viewed along the c axis showing the ribbon like structure with intra-ribbon C—H···O hydrogen bonds.

## supplementary crystallographic information

### 1. Comment

Anti-inflammatory drugs are widely prescribed in clinical practice to treat a broad range of diseases associated with inflammatory processes (Merlet *et al*., 2013). Naproxen, (*S*)-(+)-6-methoxy-a-methyl-2-naphthaleneacetic acid, is a non-steroidal anti-inflammatory drug used in painful inflammatory rheumatic and certain non-rheumatic conditions (Khanna *et al.*, 2006; Bhaduri *et al.*, 1995; Dharmani *et al.*, 2004). As with other common non-steroidal anti-inflammatory drugs (NSAIDs), Naproxen has been reported to be associated with a number of undesirable effects, which in particular include gastrointestinal (GI) toxicity (Neeraj *et al.*, 2010). These reports confirm that gastrointestinal side-effects are due to the presence of a free carboxylic group (Asif 2009). Therefore, temporary masking or manipulation of the acidic group in NSAIDs are promising means to reduce or to abolish the GI toxicity due to the local action mechanism (Parmeshwari *et al.*, 2009). Based on such facts, the title compound has been prepared.

In the title molecule (Fig. 1), the naphthalene ring system (C2–C11) is essentially planar with an r.m.s. deviation of 0.003 Å and makes a dihedral angle of 77.57 (12)° with the terminal phenyl ring (C16–C21).

In the crystal structure, the molecules exist in the "extended" form. The packing consists of ribbons of molecules extending parallel to *c* (Fig. 2) and associated *via* N—H···O and weak C—H···O hydrogen bonds (Table 1 and Fig. 3). In addition, C—H···p interactions are observed (Table 1).

### 2. Experimental

A mixture of 244 mg (1 mmol) of 2-(6-methoxynaphthalen-2-yl)propanehydrazide and benzaldehyde 106 mg (1 mmol) in 30 ml ethanol with few drops of glacial acetic acid as a catalyst was refluxed for 5 h. After the reaction mixture was cooled to ambient temperature, the excess solvent was evaporated under vacuum and the resulting solid product was filtered off, washed with cold ethanol and recrystallized from ethanol to afford high quality, clear colourless blocks (*M*.p. 453 – 455 K) in a good yield 79%..

### 3. Refinement

The amino H atom was located in a difference Fourier map and was refined with *U*_iso_(H) = 1.2*U*_eq_(N). C-bound H atoms were positioned geometrically and allowed to ride on their parent atoms with C—H = 0.95 - 1.00 Å, with *U*_iso_(H) = 1.2 or 1.5*U*_iso_(C).

### Crystal data

**Table:**

C_21_H_20_N_2_O_2_	*F*(000) = 704
*M**_r_* = 332.39	*D*_x_ = 1.293 Mg m^−^^3^
Orthorhombic, *P*2_1_2_1_2_1_	Mo *K*α radiation, λ = 0.71073 Å
Hall symbol: P 2ac 2ab	Cell parameters from 9954 reflections
*a* = 10.3754 (17) Å	θ = 2.3–28.2°
*b* = 32.519 (5) Å	µ = 0.08 mm^−^^1^
*c* = 5.0615 (8) Å	*T* = 150 K
*V* = 1707.7 (5) Å^3^	Block, clear colourless
*Z* = 4	0.15 × 0.11 × 0.11 mm

### Data collection

**Table:**

Bruker SMART APEX CCD diffractometer	4230 independent reflections
Radiation source: fine-focus sealed tube	3882 reflections with *I* > 2σ(*I*)
Graphite monochromator	*R*_int_ = 0.053
Detector resolution: 8.3660 pixels mm^-1^	θ_max_ = 28.3°, θ_min_ = 2.1°
φ and ω scans	*h* = −13→13
Absorption correction: multi-scan *SADABS* (Bruker, 2013)	*k* = −43→42
*T*_min_ = 0.73, *T*_max_ = 0.99	*l* = −6→6
28152 measured reflections	

### Refinement

**Table:**

Refinement on *F*^2^	Primary atom site location: structure-invariant direct methods
Least-squares matrix: full	Secondary atom site location: difference Fourier map
*R*[*F*^2^ > 2σ(*F*^2^)] = 0.052	H atoms treated by a mixture of independent and constrained refinement
*wR*(*F*^2^) = 0.128	*w* = 1/[Σ^2^(*F*_o_^2^) + (0.0416*P*)^2^ + 1.3067*P*] where *P* = (*F*_o_^2^ + 2*F*_c_^2^)/3
*S* = 1.10	(Δ/σ)_max_ < 0.001
4230 reflections	Δρ_max_ = 0.34 e Å^−^^3^
232 parameters	Δρ_min_ = −0.26 e Å^−^^3^
0 restraints	

### Special details

**Table:**

Experimental. The diffraction data were obtained from 3 sets of 400 frames, each of width 0.5° in ω, colllected at φ = 0.00, 90.00 and 180.00° and 2 sets of 800 frames, each of width 0.45° in φ, collected at ω = -30.00 and 210.00°. The scan time was 25 sec/frame.
Geometry. Bond distances, angles *etc*. have been calculated using the rounded fractional coordinates. All su's are estimated from the variances of the (full) variance-covariance matrix. The cell e.s.d.'s are taken into account in the estimation of distances, angles and torsion angles
Refinement. Refinement on *F*^2^ for ALL reflections except those flagged by the user for potential systematic errors. Weighted *R*-factors *wR* and all goodnesses of fit *S* are based on *F*^2^, conventional *R*-factors *R* are based on *F*, with *F* set to zero for negative *F*^2^. The observed criterion of *F*^2^ > σ(*F*^2^) is used only for calculating -*R*-factor-obs *etc*. and is not relevant to the choice of reflections for refinement. *R*-factors based on *F*^2^ are statistically about twice as large as those based on *F*, and *R*-factors based on ALL data will be even larger.

### Fractional atomic coordinates and isotropic or equivalent isotropic displacement parameters (Å^2^)

**Table:**

	*x*	*y*	*z*	*U*_iso_*/*U*_eq_	
O1	0.8923 (2)	0.05817 (7)	1.2997 (5)	0.0341 (7)	
O2	1.0001 (3)	0.28050 (6)	0.4958 (4)	0.0327 (7)	
N1	0.9781 (2)	0.29446 (7)	0.0582 (5)	0.0212 (7)	
N2	0.9700 (2)	0.33651 (7)	0.0988 (5)	0.0217 (6)	
C1	0.7617 (3)	0.05673 (11)	1.3868 (7)	0.0360 (10)	
C2	0.9231 (3)	0.08584 (8)	1.1061 (6)	0.0251 (8)	
C3	0.8391 (3)	0.11238 (8)	0.9855 (6)	0.0236 (8)	
C4	0.8842 (3)	0.13987 (8)	0.7876 (6)	0.0206 (8)	
C5	0.8006 (3)	0.16752 (9)	0.6535 (6)	0.0233 (8)	
C6	0.8471 (3)	0.19367 (8)	0.4643 (6)	0.0234 (8)	
C7	0.9800 (3)	0.19449 (8)	0.3953 (6)	0.0206 (7)	
C8	1.0616 (3)	0.16783 (8)	0.5222 (6)	0.0222 (7)	
C9	1.0170 (3)	0.13986 (8)	0.7169 (6)	0.0213 (7)	
C10	1.1001 (3)	0.11197 (9)	0.8480 (6)	0.0255 (8)	
C11	1.0553 (3)	0.08558 (9)	1.0365 (6)	0.0270 (8)	
C12	1.0243 (3)	0.22433 (8)	0.1836 (6)	0.0216 (7)	
C13	1.1682 (3)	0.22119 (9)	0.1119 (7)	0.0282 (8)	
C14	0.9980 (3)	0.26884 (8)	0.2669 (5)	0.0203 (7)	
C15	0.9435 (3)	0.35759 (8)	−0.1088 (6)	0.0222 (8)	
C16	0.9384 (3)	0.40269 (8)	−0.0976 (6)	0.0222 (7)	
C17	0.9991 (3)	0.42472 (9)	0.1062 (6)	0.0261 (8)	
C18	0.9965 (3)	0.46725 (9)	0.1063 (7)	0.0307 (9)	
C19	0.9334 (3)	0.48871 (9)	−0.0913 (7)	0.0316 (9)	
C20	0.8714 (3)	0.46720 (10)	−0.2899 (7)	0.0316 (9)	
C21	0.8748 (3)	0.42453 (10)	−0.2946 (7)	0.0286 (9)	
H1	0.976 (3)	0.2849 (10)	−0.117 (7)	0.025 (8)*	
H1A	0.73540	0.08400	1.44880	0.0540*	
H1B	0.75370	0.03690	1.53170	0.0540*	
H1C	0.70610	0.04830	1.23990	0.0540*	
H3	0.75070	0.11240	1.03410	0.0280*	
H5	0.71140	0.16790	0.69570	0.0280*	
H6	0.78920	0.21170	0.37680	0.0280*	
H8	1.15060	0.16810	0.47840	0.0270*	
H10	1.18900	0.11160	0.80360	0.0310*	
H11	1.11280	0.06710	1.12110	0.0320*	
H12	0.97320	0.21870	0.02000	0.0260*	
H13A	1.22050	0.22710	0.26860	0.0420*	
H13B	1.18840	0.24110	−0.02710	0.0420*	
H13C	1.18720	0.19340	0.04850	0.0420*	
H15	0.92710	0.34390	−0.27110	0.0270*	
H17	1.04190	0.41040	0.24420	0.0310*	
H18	1.03870	0.48190	0.24370	0.0370*	
H19	0.93260	0.51790	−0.09050	0.0380*	
H20	0.82620	0.48170	−0.42390	0.0380*	
H21	0.83320	0.41010	−0.43380	0.0340*	

### Atomic displacement parameters (Å^2^)

**Table:**

	*U*^11^	*U*^22^	*U*^33^	*U*^12^	*U*^13^	*U*^23^
O1	0.0352 (12)	0.0320 (12)	0.0351 (12)	0.0008 (10)	0.0024 (10)	0.0129 (10)
O2	0.0526 (15)	0.0248 (10)	0.0207 (10)	−0.0008 (10)	0.0018 (10)	−0.0023 (8)
N1	0.0266 (12)	0.0207 (11)	0.0162 (11)	−0.0018 (9)	−0.0001 (9)	−0.0013 (9)
N2	0.0218 (11)	0.0211 (11)	0.0223 (11)	−0.0016 (9)	−0.0007 (10)	−0.0015 (9)
C1	0.0376 (17)	0.0389 (18)	0.0314 (17)	−0.0073 (14)	0.0031 (15)	0.0077 (15)
C2	0.0324 (15)	0.0198 (13)	0.0230 (13)	−0.0012 (11)	0.0018 (12)	0.0017 (11)
C3	0.0209 (13)	0.0216 (13)	0.0283 (15)	−0.0007 (10)	0.0017 (11)	0.0002 (11)
C4	0.0220 (13)	0.0177 (13)	0.0220 (13)	0.0001 (10)	0.0000 (11)	−0.0012 (10)
C5	0.0176 (12)	0.0234 (13)	0.0290 (16)	0.0029 (10)	0.0003 (11)	0.0017 (11)
C6	0.0217 (13)	0.0218 (13)	0.0267 (15)	0.0039 (10)	−0.0012 (11)	0.0020 (11)
C7	0.0223 (12)	0.0176 (12)	0.0218 (12)	−0.0002 (9)	0.0028 (11)	−0.0017 (10)
C8	0.0179 (12)	0.0245 (13)	0.0242 (13)	0.0000 (10)	0.0020 (11)	−0.0023 (11)
C9	0.0215 (13)	0.0197 (12)	0.0227 (13)	0.0011 (10)	−0.0004 (11)	−0.0017 (10)
C10	0.0200 (13)	0.0293 (15)	0.0271 (16)	0.0055 (11)	0.0002 (12)	0.0004 (11)
C11	0.0296 (15)	0.0272 (14)	0.0243 (15)	0.0073 (11)	−0.0042 (12)	0.0033 (11)
C12	0.0228 (13)	0.0206 (12)	0.0213 (13)	−0.0018 (10)	0.0009 (11)	−0.0015 (10)
C13	0.0259 (14)	0.0268 (14)	0.0319 (16)	−0.0001 (11)	0.0069 (13)	0.0007 (13)
C14	0.0208 (13)	0.0236 (13)	0.0164 (12)	−0.0027 (10)	0.0034 (10)	−0.0014 (10)
C15	0.0203 (12)	0.0257 (14)	0.0205 (13)	−0.0023 (10)	0.0002 (11)	−0.0021 (11)
C16	0.0183 (12)	0.0242 (13)	0.0241 (13)	−0.0002 (10)	0.0025 (11)	0.0009 (11)
C17	0.0247 (14)	0.0271 (14)	0.0265 (13)	0.0003 (11)	−0.0023 (12)	0.0003 (12)
C18	0.0335 (16)	0.0269 (15)	0.0316 (15)	−0.0025 (12)	0.0001 (14)	−0.0045 (12)
C19	0.0348 (17)	0.0204 (14)	0.0397 (18)	0.0039 (11)	0.0070 (15)	0.0009 (13)
C20	0.0292 (16)	0.0324 (17)	0.0333 (16)	0.0065 (12)	−0.0008 (13)	0.0066 (13)
C21	0.0251 (14)	0.0320 (16)	0.0286 (15)	−0.0002 (11)	−0.0035 (12)	0.0013 (13)

### Geometric parameters (Å, º)

**Table:**

O1—C1	1.426 (4)	C17—C18	1.383 (4)
O1—C2	1.368 (4)	C18—C19	1.384 (5)
O2—C14	1.219 (3)	C19—C20	1.383 (5)
N1—N2	1.385 (3)	C20—C21	1.388 (5)
N1—C14	1.361 (4)	C1—H1A	0.9800
N2—C15	1.284 (4)	C1—H1B	0.9800
N1—H1	0.94 (3)	C1—H1C	0.9800
C2—C11	1.416 (4)	C3—H3	0.9500
C2—C3	1.370 (4)	C5—H5	0.9500
C3—C4	1.422 (4)	C6—H6	0.9500
C4—C9	1.424 (4)	C8—H8	0.9500
C4—C5	1.422 (4)	C10—H10	0.9500
C5—C6	1.369 (4)	C11—H11	0.9500
C6—C7	1.423 (4)	C12—H12	1.0000
C7—C12	1.517 (4)	C13—H13A	0.9800
C7—C8	1.372 (4)	C13—H13B	0.9800
C8—C9	1.419 (4)	C13—H13C	0.9800
C9—C10	1.416 (4)	C15—H15	0.9500
C10—C11	1.365 (4)	C17—H17	0.9500
C12—C13	1.540 (4)	C18—H18	0.9500
C12—C14	1.532 (4)	C19—H19	0.9500
C15—C16	1.469 (4)	C20—H20	0.9500
C16—C21	1.391 (4)	C21—H21	0.9500
C16—C17	1.405 (4)		
			
C1—O1—C2	117.7 (2)	O1—C1—H1B	109.00
N2—N1—C14	119.9 (2)	O1—C1—H1C	110.00
N1—N2—C15	114.7 (2)	H1A—C1—H1B	109.00
C14—N1—H1	122 (2)	H1A—C1—H1C	109.00
N2—N1—H1	118 (2)	H1B—C1—H1C	109.00
O1—C2—C11	113.6 (3)	C2—C3—H3	120.00
O1—C2—C3	125.8 (3)	C4—C3—H3	120.00
C3—C2—C11	120.6 (3)	C4—C5—H5	120.00
C2—C3—C4	120.0 (3)	C6—C5—H5	120.00
C5—C4—C9	118.1 (3)	C5—C6—H6	119.00
C3—C4—C9	119.7 (3)	C7—C6—H6	119.00
C3—C4—C5	122.2 (3)	C7—C8—H8	119.00
C4—C5—C6	120.8 (3)	C9—C8—H8	119.00
C5—C6—C7	121.7 (3)	C9—C10—H10	119.00
C6—C7—C12	118.6 (3)	C11—C10—H10	119.00
C6—C7—C8	118.1 (3)	C2—C11—H11	120.00
C8—C7—C12	123.2 (3)	C10—C11—H11	120.00
C7—C8—C9	122.0 (3)	C7—C12—H12	108.00
C4—C9—C10	118.1 (3)	C13—C12—H12	108.00
C4—C9—C8	119.4 (3)	C14—C12—H12	108.00
C8—C9—C10	122.5 (3)	C12—C13—H13A	109.00
C9—C10—C11	121.5 (3)	C12—C13—H13B	109.00
C2—C11—C10	120.0 (3)	C12—C13—H13C	109.00
C13—C12—C14	107.5 (2)	H13A—C13—H13B	109.00
C7—C12—C14	110.9 (2)	H13A—C13—H13C	110.00
C7—C12—C13	114.7 (2)	H13B—C13—H13C	109.00
O2—C14—C12	123.5 (2)	N2—C15—H15	120.00
O2—C14—N1	123.4 (2)	C16—C15—H15	120.00
N1—C14—C12	113.1 (2)	C16—C17—H17	120.00
N2—C15—C16	120.6 (3)	C18—C17—H17	120.00
C15—C16—C17	121.4 (3)	C17—C18—H18	120.00
C17—C16—C21	118.6 (3)	C19—C18—H18	120.00
C15—C16—C21	120.0 (3)	C18—C19—H19	120.00
C16—C17—C18	120.1 (3)	C20—C19—H19	120.00
C17—C18—C19	120.9 (3)	C19—C20—H20	120.00
C18—C19—C20	119.4 (3)	C21—C20—H20	120.00
C19—C20—C21	120.4 (3)	C16—C21—H21	120.00
C16—C21—C20	120.7 (3)	C20—C21—H21	120.00
O1—C1—H1A	110.00		
			
C1—O1—C2—C3	−0.5 (4)	C6—C7—C12—C13	−176.7 (3)
C1—O1—C2—C11	178.8 (3)	C6—C7—C12—C14	61.4 (3)
C14—N1—N2—C15	−176.1 (3)	C8—C7—C12—C13	2.6 (4)
N2—N1—C14—O2	5.6 (4)	C8—C7—C12—C14	−119.4 (3)
N2—N1—C14—C12	−171.5 (2)	C7—C8—C9—C4	−1.3 (4)
N1—N2—C15—C16	−176.9 (2)	C7—C8—C9—C10	179.5 (3)
O1—C2—C3—C4	179.6 (3)	C4—C9—C10—C11	0.2 (4)
C11—C2—C3—C4	0.3 (4)	C8—C9—C10—C11	179.4 (3)
O1—C2—C11—C10	−179.2 (3)	C9—C10—C11—C2	−0.4 (5)
C3—C2—C11—C10	0.1 (4)	C7—C12—C14—O2	31.6 (4)
C2—C3—C4—C5	178.9 (3)	C7—C12—C14—N1	−151.3 (3)
C2—C3—C4—C9	−0.4 (4)	C13—C12—C14—O2	−94.4 (4)
C3—C4—C5—C6	179.9 (3)	C13—C12—C14—N1	82.6 (3)
C9—C4—C5—C6	−0.8 (4)	N2—C15—C16—C17	20.1 (5)
C3—C4—C9—C8	−179.0 (3)	N2—C15—C16—C21	−161.3 (3)
C3—C4—C9—C10	0.2 (4)	C15—C16—C17—C18	177.7 (3)
C5—C4—C9—C8	1.6 (4)	C21—C16—C17—C18	−0.9 (5)
C5—C4—C9—C10	−179.2 (3)	C15—C16—C21—C20	−178.6 (3)
C4—C5—C6—C7	−0.4 (4)	C17—C16—C21—C20	0.0 (5)
C5—C6—C7—C8	0.7 (4)	C16—C17—C18—C19	0.7 (5)
C5—C6—C7—C12	180.0 (3)	C17—C18—C19—C20	0.5 (5)
C6—C7—C8—C9	0.2 (4)	C18—C19—C20—C21	−1.5 (5)
C12—C7—C8—C9	−179.1 (3)	C19—C20—C21—C16	1.2 (5)

### Hydrogen-bond geometry (Å, º)

Cg2 and Cg3 are the centroids of the C4–C9 benzene and C16–C21 phenyl rings, respectively.

**Table:**

*D*—H···*A*	*D*—H	H···*A*	*D*···*A*	*D*—H···*A*
N1—H1···O2^i^	0.94 (3)	1.98 (4)	2.892 (3)	163 (3)
C15—H15···O2^i^	0.95	2.49	3.261 (3)	138
C18—H18···O1^ii^	0.95	2.59	3.209 (4)	123
C1—H1*C*···*Cg*3^iii^	0.98	2.91	3.696 (3)	138
C12—H12···*Cg*2^i^	1.00	2.78	3.661 (3)	147

Symmetry codes: (i) *x*, *y*, *z*−1; (ii) −*x*+2, *y*+1/2, −*z*+3/2; (iii) *x*−1/2, −*y*+1/2, −*z*+1.
